# Impact of surgical margins on local recurrence rates in breast-conserving surgery

**DOI:** 10.6026/973206300211631

**Published:** 2025-06-30

**Authors:** Rakshana Munusamy, Mohammed Mohsen Abdel Raouf, Shakthiyan Gopal, J. Naveen Bose, Manisha Mohammed Shaan, Kaushik J.

**Affiliations:** 1 Department of Surgery, Madurai Medical College, Madurai, Tamil Nadu, India; 2 Department of Internal Medicine, NUB NAHDA University Hospital, Banisuef, Egypt; 3 Department of Neuro Surgery, Sagar Hospital, Bengaluru, Karnataka, India; 4 Department of Emergency Medicine, Madurai Medical College, Madurai, Tamil Nadu, India; 5 Department of Medical Oncology, SRM Medical College, Chennai, Tamil Nadu, India; 6 Department of Surgical Oncology, Rajiv Gandhi Cancer Institute & Research Centre, New Delhi, India

**Keywords:** Breast-conserving surgery (BCS), surgical margins, local recurrence, breast cancer, oncological safety, tumor resection

## Abstract

A conservative surgical breast intervention is broadly acceptable as an appropriate treatment option in preliminary stages of breast cancer and is recommended in order to establish oncologic safety while achieving aesthetics of the breast. Therefore, it is of interest to follow-up for five years among 150 patients who received breast-conserving surgery with an understanding of surgical margin status which might be reported positive, close, or negative; the mean incidence of overall local recurrence is estimated at 8%. Higher values, however, were recorded in positive margin patients of 25%. Close and negative margins were significantly at 10% and 5%, respectively. Tumour size and grade, with the margin, are significant indicators of recurrence of p <0.05. In such findings lies the critical relevance of achieving the surgical margins clean in order to minimize the percentage of local recurrences and have better long term results in a patient with carcinoma breast.

## Background:

Breast-conserving surgery, which is also known as lumpectomy or wide local excision, has emerged as a cornerstone of treatment for early-stage breast cancer [1]. It aims at ensuring complete removal of the tumor with preserved breast aesthetics and is therefore an alternative to mastectomy with comparable long-term survival rates combined with appropriate adjuvant therapies [2]. However, attainment of clear surgical margins remains extremely critical to achieve the anticipated optimal oncologic outcomes and relies heavily on the success of BCS [3]. Evolution of the concept of adequate margin has also followed a course. Recent guidelines emphasize that "no ink on tumor" is an acceptable criterion for negative margins [4]. Although these advances have occurred, the margins may still be positive or close and it is known that a positive margin carry a greater risk of recurrence with the potential for re-excision or adjuvant therapies [5]. Practically, the tumor size, grade, histological subtype, among others, affects the chances of achieving negative margins [6]. This is a prospective study whose purpose is to monitor the impacts of surgical margin status on local recurrence rates in patients who have undergone breast-conserving surgery [7]. The authors analyze the patterns of recurrence and associated factors for a five-year follow-up period and seek to improve understanding of how best to optimize surgical techniques and thus improve outcomes in breast cancer [8]. Therefore it is of interest to Impact of surgical margins on local recurrence rates in breast-conserving surgery.

## Materials and Methods:

This prospective study was conducted at a tertiary care center over a period of five years. A total of 150 female patients diagnosed with early-stage breast cancer (stages I and II) and undergoing breast-conserving surgery (BCS) were enrolled. Ethical approval was obtained from the institutional review board and all participants provided informed consent prior to inclusion. Patients were included based on a confirmed histological diagnosis of invasive carcinoma or ductal carcinoma in situ (DCIS) and excluded if they had metastatic disease, prior breast surgery, or were unfit for follow-up. Surgical margins were classified intra-operatively as positive (tumor at inked margin), close (tumor within 1 mm of the margin), or negative (no tumor within 1 mm of the margin). Clinical, histopathological and imaging data was collected preoperatively that includes tumor size, grade and lymph node status and receptor profile. All the patients received adjuvant therapy on the recommendations from the multidisciplinary teams with radiation, chemotherapy, or endocrine therapy. Follow-up was done every six months for the first three years and then annually to check for local recurrence. Recurrence was defined as the reappearance of cancer at the original surgical site confirmed by imaging and histopathology. Statistical analysis was performed using SPSS version 25 and logistic regression and Kaplan-Meier survival curves were used to evaluate recurrence risk factors, considering p < 0.05 as significant.

## Results:

This study evaluated 150 patients undergoing breast-conserving surgery (BCS) for early-stage breast cancer. The mean age of the patients was 52.4 years, with the majority (65%) presenting with stage I disease. Surgical margins were categorized as negative in 72%, close in 18% and positive in 10% of cases. The overall local recurrence rate was 8% during the five-year follow-up period. Recurrence rates were significantly higher in patients with positive margins (25%) compared to those with close (10%) or negative margins (5%) (p < 0.05). Kaplan-Meier survival analysis demonstrated significantly shorter recurrence-free survival in patients with positive margins. Table 1 (see PDF) highlights the demographic and clinical characteristics of the study participants, showing a predominance of stage I disease and estrogen receptor-positive (ER+) tumors. Table 2 (see PDF) shows the distribution of surgical margin status among the study population, highlighting that 72% of cases achieved negative margins. Table 3 (see PDF) compares the recurrence rates based on margin status, indicating a significantly higher recurrence rate in patients with positive margins. Table 4 (see PDF) illustrates the distribution of recurrence rates based on tumor size, showing that larger tumors (>2 cm) were associated with higher recurrence rates. Table 5 (see PDF) highlights the correlation between tumor grade and recurrence, with high-grade tumors showing significantly higher recurrence rates (p < 0.05). Table 6 (see PDF) shows the Kaplan-Meier recurrence-free survival analysis, indicating shorter survival times for patients with positive margins compared to those with negative or close margins. Table 7 (see PDF) describes the adjuvant therapy distribution among the study population, showing high rates of chemotherapy and radiation therapy in patients with positive margins. Table 8 (see PDF) compares recurrence rates based on HER2 receptor status, indicating higher rates in HER2-positive tumors. Table 9 (see PDF) examines recurrence rates based on lymph node involvement, showing that positive lymph nodes were associated with significantly higher recurrence rates. Table 10 (see PDF) summarizes recurrence rates in patients with different combinations of margin status and adjuvant therapy, highlighting that combined chemotherapy and radiation therapy significantly reduced recurrence rates. The study emphasizes the importance of achieving adequate surgical margins in breast-conserving surgery (BCS) to minimize recurrence. Table 1 (see PDF) shows a mean patient age of 52.4 years with most cases at stage I. Table 2 (see PDF) highlights that 72% achieved negative margins. Recurrence rates (Table 3 - see PDF) were highest in positive margins (25%), compared to close (10%) and negative (5%). Larger tumors (>2 cm) and high-grade tumors had higher recurrence rates (15% and 18%, respectively, as shown in Tables 4 - see PDF and 5 - see PDF). Kaplan-Meier analysis (Table 6 - see PDF) revealed shorter recurrence-free survival in positive-margin patients. Table 7 - see PDF links positive margins to increased use of adjuvant therapy, while Table 8 - see PDF associates 18% of recurrences with HER2-positive tumors. Lymph node positivity was tied to a 20% recurrence rate (Table 9 - see PDF). Table 10 (see PDF) underscores the combined impact of margin status and adjuvant therapy, reinforcing that achieving negative margins is crucial for better outcomes.

## Discussion:

This study focuses on the role of surgical margins in the determination of the local recurrence rate in BCS. Although an overall recurrence rate of 8% was found, the results indicated a recurrence rate is significantly higher at positive margins at 25%, with close margins at 10% and at negative margins with 5% as p was < 0.05 [9]. Such results further emphasize that clear surgical margins are crucial for diminished recurrence risk during BCS as well as improved outcomes in the long term. Tumor size, grade and lymph node involvement were all significantly associated with recurrence. More significant tumors (>2 cm) and high-grade tumors were associated with higher recurrence and there is a need to have proper planning before surgery for these cases as well as assessing margins [10]. HER2-positive tumors are associated with increased recurrence rates underlining the role of tumor biology in recurrence risk and the importance of targeted therapy. Adjuvant therapy, including radiation therapy and chemotherapy, revealed a high percentage of recurrence prevention in patients with close or positive margins [11]. Patients whose margins were negative were well differentiated, with a higher recurrence-free survival compared to patients whose margins were either close or positive according to the Kaplan-Meier analysis [12]. Such findings are in accordance with the existing literature advocating for a multidisciplinary approach, combining precise surgical techniques with adjuvant therapies and follow-up. Intraoperative margin assessment techniques may involve frozen sections or cavity shaving to improve margin clearance in BCS [13]. Further studies should target new imaging modalities and molecular markers that help predict recurrence risk and provide focused surgical and adjuvant interventions accordingly.

## Conclusion:

Wide margins at the time of operation are significant in reducing local recurrence after breast-conserving surgery. Patients with positive margins had a recurrence rate of 25%, compared to 10% for close and 5% for negative margins. Size, grade, lymph node status, and HER2 status also impact the risk of recurrence. Achieving negative margins through careful surgery, intraoperative assessment, and multidisciplinary therapy matters in improving precision-free survival and overall results in patients with breast cancer.

## References

[1] Corradini S (2019). Cancers (Basel)..

[2] https://pubmed.ncbi.nlm.nih.gov/30725899/.

[3] Chae S (2022). Curr Oncol..

[4] Pilewskie M (2018). Cancer..

[5] Orosco R.K (2018). Sci Rep..

[6] Sambri A (2021). Cancers (Basel)..

[7] Di Lena E (2024). Ann Surg Oncol..

[8] Goel A (2021). Indian J Surg Oncol..

[9] Houssami N (2014). Ann Surg Oncol..

[10] Pop C.F (2025). NPJ Breast Cancer..

[11] Yang J (2021). Comput Struct Biotechnol J..

[12] Stojadinovic A (2002). Ann Surg..

[13] Li W (2022). Gland Surg..

